# Investigation of the Photocatalytic Activity of Copper-Modified Commercial Titania (P25) in the Process of Carbon Dioxide Photoreduction

**DOI:** 10.3390/ma17246139

**Published:** 2024-12-15

**Authors:** Konrad Sebastian Sobczuk, Iwona Pełech, Daniel Sibera, Piotr Staciwa, Agnieszka Wanag, Ewa Ekiert, Joanna Kapica-Kozar, Katarzyna Ćmielewska, Ewelina Kusiak-Nejman, Antoni Waldemar Morawski, Urszula Narkiewicz

**Affiliations:** 1 Department of Inorganic Chemical Technology and Environment Engineering, Faculty of Chemical Technology and Engineering, West Pomeranian University of Technology in Szczecin, Pułaskiego 10, 70-322 Szczecin, Poland; daniel.sibera@zut.edu.pl (D.S.); piotr.staciwa@zut.edu.pl (P.S.); agnieszka.wanag@zut.edu.pl (A.W.); ewa.dabrowa@zut.edu.pl (E.E.); joanna.kapica@zut.edu.pl (J.K.-K.); k.cmielewska@icloud.com (K.Ć.); ekusiak@zut.edu.pl (E.K.-N.); antoni.morawski@zut.edu.pl (A.W.M.); urszula.narkiewicz@zut.edu.pl (U.N.); 2 Department of Construction and Road Engineering, Faculty of Civil and Environmental Engineering, West Pomeranian University of Technology in Szczecin, Piastów 50a, 70-311 Szczecin, Poland

**Keywords:** carbon dioxide, copper modification, microwave-assisted solvothermal processing, photocatalytic reduction, photocatalyst

## Abstract

The photocatalytic reduction of CO_2_ to useful products is an area of active research because it shows a potential to be an efficient tool for mitigating climate change. This work investigated the modification of titania with copper(II) nitrate and its impact on improving the CO_2_ reduction efficiency in a gas-phase batch photoreactor under UV–Vis irradiation. The investigated photocatalysts were prepared by treating P25-copper(II) nitrate suspensions (with various Cu^2+^ concentrations), alkalized with ammonia water, in a microwave-assisted solvothermal reactor. The titania-based photocatalysts were characterized by SEM, EDS, ICP-OES, XRD and UV-Vis/DR methods. Textural properties were measured by the low-temperature nitrogen adsorption/desorption studies at 77 K. P25 photocatalysts modified with copper(II) nitrate used in the process of carbon dioxide reduction allowed for a higher efficiency both for the photocatalytic reduction of CO_2_ to CH_4_ and for the photocatalytic water decomposition to hydrogen as compared to a reference. Similarly, modified samples showed significantly higher selectivity towards methane in the CO_2_ conversion process than the unmodified sample (a change from 30% for a reference sample to 82% for the P25-R-Cu-0.1 sample after the 6 h process). It was found that smaller loadings of Cu are more beneficial for increasing the photocatalytic activity of a sample.

## 1. Introduction

Hydrocarbons made from crude oil serve many functions (including as energy sources, chemical feedstock, chemical reducing agents, etc.), which makes them incredibly important for the global economy [1]. Unfortunately, the combustion of hydrocarbon-based fuels and materials has a significant drawback of environmental pollution, which includes carbon dioxide emissions [2]. With CO_2_ being the predominant greenhouse gas responsible for global warming [3] and climate change being a major concern for humanity, reducing the levels of CO_2_ emitted to the atmosphere becomes an important issue for all communities.

However, a pathway to decreasing carbon dioxide levels cannot be realistically based on ceasing to use oils and other hydrocarbons, because of their importance in maintaining improved living standards and energy demand during economic and populational growth [4,5]. In order to create a sustainable model for the economy, alternatives which reuse CO_2_ to produce other marketable products are taken into consideration [6]. Carbon capture and utilization (CCU) methods have the potential to become an important tool for mitigating negative aspects of climate change [7].

One of the available routes of converting carbon dioxide (as an alternative to capturing carbon dioxide and burying it underground for long-term storage [6]) is based on the photo-driven reduction of CO_2_ into fuels and/or platform chemicals using heterogeneous photocatalysis [3]. The possibility of using UV and/or visible light as excitation sources for the electrons in the valence band (VB) of semiconductor catalysts suggests that the photocatalytic conversion of CO_2_ with H_2_O may be a promising technique to utilize carbon dioxide [5]. Possible reaction directions allow for the photoreduction of CO_2_ on the catalyst surface to produce energy-bearing products such as methane (CH_4_) [8,9], methanol (CH_3_OH) [10,11], carbon monoxide (CO) [12,13], formic acid (HCOOH) [14,15] and formaldehyde (HCHO) [16,17]. The introduction of water into the system also allows one to obtain hydrogen (H_2_) created as a result of the water splitting [18,19].

Widely used photocatalytically active materials include those based on titanium dioxide [20,21] because this approach allows one to obtain previously described products [3], has a high resistance to photocorrosion and has long-term stability while being a low-cost material [19,22]. These characteristics render these materials a promising tool for mitigating rising CO_2_ levels.

Titanium dioxide is an environmentally friendly semiconductor [23], which implies that its functions may be explained via the band theory [24,25]. After absorbing photons of energy larger than the band gap (E_g_) of TiO_2_, electrons from the valence band (VB) are excited to the conduction band (CB), which creates electron (e^−^)–hole (h^+^) pairs in the material. These charge carriers have the ability to quickly travel to the surfaces of catalyst particles, where they can take part in redox reactions if suitable substrates are available [26].

Pure titanium dioxide has a quite large band gap of E_g_~3.1 eV [27], which makes it predominantly active under ultraviolet (UV) light, because of its photons’ energy being greater than the band gap of TiO_2_ [28,29]. This characteristic of the material leads researchers to modify this material to decrease the E_g_ value, which allows for the charge carrier (e^−^–h^+^) pairs to form in a material more easily. The narrowing of this gap is often achieved via doping [28], the incorporation of d-block metal ions [30], or the introduction of other metals/metal oxides as interfaces in the structure of a photocatalyst, which allows for a considerable synergistic effect to occur between two components [31].

The icorporation of metal ions into titania can significantly extend the photocatalysts’ absorption range into the visible region [32] while simultaneously improving photocatalytic activity [30], which makes it one of the most popular methods for modifying titanium dioxide. Such modifications are often executed using copper (Cu) [11,30,33], due to its role as an electron scavenger in the material. It has been reported that the successful addition of Cu into TiO_2_ enhances its activity by creating a new energy level below the conduction band of TiO_2_, which can act as an electron trapper [34].

Additionally, modification with copper frequently allows for the creation of copper(II) oxide (CuO) [35], which also exhibits semiconductor properties and can be used as a photocatalyst [36]. It has been reported that the introduction of surface/interfacial Cu^2+^ sites to CuO−TiO_2_ nanocomposites improves the material photocatalytic activity [31], mostly due to the existence of a particular nanostructured morphology of interwoven rutile and anatase crystallites, which promotes spatial charge separation [37]. This improved photocatalytic activity of copper can be beneficial in various applications, such as H_2_ production, CO_2_ reduction and pollutant treatment [34].

Multiple studies have mentioned the heightened photocatalytic activity of samples modified with copper [38,39,40,41]. Zhang et al. obtained a material based on P25 loaded with copper 0.4 wt.%, which allowed them to obtain a formation rate of methane equal to 8 μmol·g_material_^−1^·h^−1^ [40]. Similarly, Neatu et al. [38] reported that selectivity of CO_2_ conversion towards methane is high (71.4%) with a formation rate of methane of 40 μmol·g_material_^−1^·h^−1^, which suggest that modification of P25 with copper leads to a powder catalyst with high potential for carbon dioxide reduction.

This study aims to investigate how modifying commercial titania P25 with differently concentrated, alkalized, copper(II) nitrate solutions using a microwave-assisted solvothermal reactor impacts its photocatalytic activity in carbon dioxide photoreduction under UV-Vis irradiation.

## 2. Results and Discussion

### 2.1. Material Characterization

#### 2.1.1. ICP (Inductively Coupled Plasma) Spectroscopy Measurements

Results for the ICP measurements for the P25 catalysts modified using copper(II) nitrate are presented in Table 1.

By comparing the assumed content of copper with that measured using instrumental methods, it can be concluded that in the presented samples various amounts of copper were identified. The overall trend of the actual concentrations growing when the assumed copper concentrations are higher is correct. There can also be found a significant correlation between the assumed copper concentrations and actual (measured by ICP) copper concentrations. For example, the actual copper concentration for sample P25-R-Cu-12 is only 1.75% different from the claimed percentage. A comparison of both presented values suggests that samples were largely successfully modified with copper in designated concentrations. Our studies have shown that the described method of modifying photocatalysts with copper compounds is an effective method for obtaining photocatalysts and, importantly, that copper is not easily washed out at various stages of material preparation.

The relative standard deviations (RSDs) of copper samples are all approximate or below 0.8%, which indicates a small deviation of each measurement from the mean, which suggests that measured values are highly repeatable.

For most samples, the actual concentration of copper in the sample is lower than the declared one, which can be explained by the method of preparation. The declared copper percentages in the names of samples are the mass concentrations of copper solutions used to modify the samples. It can be assumed that during the process there were multiple losses of copper due to the imperfect method of transferring the sample from one part of the process to another. The difference between both values of concentration can therefore be attributed to the losses during the processing of samples.

#### 2.1.2. UV-Vis/DR Spectra Measurements

**Figure 1:**
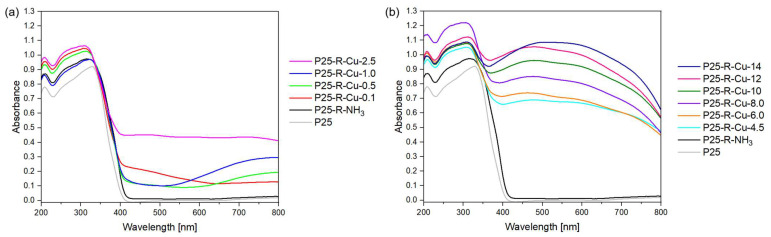
UV-Vis/DR spectra of tested photocatalysts: (**a**) reference materials and modified materials up to 2.5 wt.% Cu (theoretical); (**b**) reference materials and modified materials above the 2.5 wt.% Cu (theoretical).

Diffuse Reflectance Spectroscopy (DRS) over UV-Vis radiation was performed for the samples. The light absorption characteristics of P25, P25-R-NH_3_ and P25 with different Cu contents are demonstrated in Figure 1. Samples after simultaneous modification with ammonia water and copper(II) nitrate in a microwave-assisted solvothermal reactor exhibit strong absorption in the UV range with the absorption edge at about 385 nm due to the (characteristic for TiO_2_) intrinsic band gap absorption of titanium. Pure, unmodified, P25 does not absorb light in the visible region (400–800 nm), while significant enhancement of light absorption in this region is observed for the P25 after Cu modification.

The increases in the light absorption of the modified photocatalysts in the visible range result from the color change of samples. As the concentration of Cu increases, the color of samples changes from white to light green followed by dark green, and gray to dark brown as presented in Table 2. This type of correlation was also noted in the literature by other researchers [41,42]. Furthermore, a change in color from white to greenish is observed from sample P25-R-NH_3_ to P25-R-Cu-4.5, which agrees with another result [43].

The role of Cu modification on the curved shape of spectra is also clearly observed, which indicates the presence of various forms of Cu. The spectrum of P25-R-Cu-0.1 shows an absorption peak between 400 nm and 600 nm, while samples P25-R-Cu-0.5 and P25-R-Cu-1.0 show broad absorption peaks between 600 nm and 800 nm; both types of peaks can be attributed to the various electron transitions between the d-d orbitals of Cu^2+^, likely due to the different electronic structure around the copper ions in the sample [44,45,46,47,48]. Those peaks demonstrate the incorporation of Cu^2+^ ions into the P25 structure and the formation of a single P25:Cu phase doped by 0.1-1 wt.% of Cu (theoretical) [49]. However, for higher Cu concentrations (above 2.5 theoretical wt.% of Cu), two different bands can be observed. The first weak absorption peak in the range of 400–500 nm can be attributed to the interfacial charge transition from the TiO_2_ VB to Cu(II) clusters or CuO amorphous phase [44,45,46]. The second band located between 500 and 600 nm may be due to the presence of two additional phases, CuO and Cu_2_O, like Bensouici et al. suggests [50]. In our case, the most likely dominant phase is CuO, because XRD measurements did not show the presence of Cu_2_O; however, this does not mean that this phase is not present in a small amount, below detection.

A completely different spectrum is noted for the P25-R-Cu-2.5 sample. The broad peak in the range of 400–800 nm with three small maximums at ca. 490, 580 and 690 nm may indicate the coexistence of all reported forms [51,52].

A redshift of absorption edges into the visible light region can be attributed to the formation of the P25:Cu phase, which allows for the creation of additional energy levels beneath the conduction band (CB) in the TiO_2_, therefore lowering the overall band gap (E_g_) value. Such modification of titania with Cu might improve the absorption of UV and visible light, reduce the photoexcitation energy and decrease the recombination rate of the excited electron–hole pairs, therefore enhancing the photocatalytic activity of a material and broadening its practical applications [53,54]. In our case, the formation of those additional electronic energy levels is most likely related to the incorporation of Cu^2+^ ions within the TiO_2_ amorphous phase [31,44,55,56].

The measured band gap values are presented in Table 2. The addition of Cu reduces the band gap energy from 3.01 eV for the reference material P25-R-NH_3_ (which is lower than non-modified P25 with E_g_ = 3.24 eV) to 2.36 eV for P25-R-Cu-12. An overall trend can be observed where E_g_ decreases while the Cu concentration increases, which is in agreement with other results [45,56,57].

#### 2.1.3. XRD Measurements

**Figure 2:**
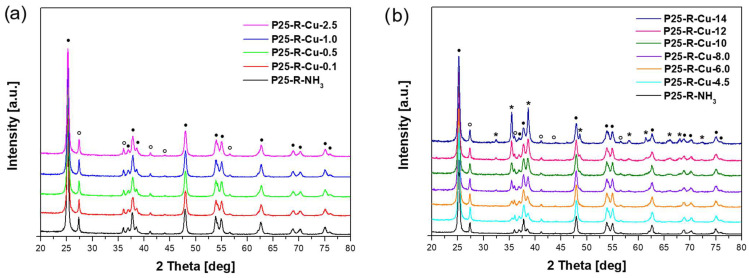
X-ray diffraction patterns of the obtained photocatalysts based on commercial titania P25 modified with copper(II) nitrate and ammonia water. Reflections attributed to anatase are marked as ●, those attributed to rutile are marked as o and those attributed to CuO are marked as *. (**a**) Reference material and modified materials up to 2.5 wt.% (theoretical); (**b**) reference material and modified materials above 2.5 wt.% (theoretical).

The phase composition of the obtained materials was investigated using the X-ray diffraction (XRD) method. In Figure 2, the diffraction patterns of obtained copper-modified P25-based photocatalysts are presented.

The XRD patterns of the photocatalysts based on commercial titanium dioxide P25 exhibit diffraction reflexes corresponding to both anatase and rutile phases. Anatase (ICDD 01-075-2547) signals were observed at around the following 2θ values: 25.3° (101), 37.0° (103), 37.8° (004), 38.6° (112), 48.1° (200), 53.9° (105), 55.1° (211), 62.7° (204), 68.8° (116), 70.3° (220), and 75.1° (215). Rutile (ICDD 01-079-6029) signals were found at the following (2θ) reflection angles: 27.4° (110), 36.1° (101), 39.2° (200), 41.2° (111), 54.3° (211), and 56.6° (220). Only for the samples modified with a high concentration of copper, above 2.5%, was the copper(II) oxide phase, CuO (ICDD 00-048-1548), also identified. For the materials with lower copper concentrations, no diffraction peaks of CuO were observed, possibly due to their low XRD detection limits. Signals related to the CuO phase were identified around the following 2θ values: 35.5° $\left(\overset{\text{¯}}{1}11\right)$, 38.7° (111), 48.7° $\left(\overset{\text{¯}}{2}02\right)$, 58.3° (202), 61.5° $\left(\overset{\text{¯}}{1}13\right)$, 65.7° (022), 66.1° $\left(\overset{\text{¯}}{3}13\right)$, and 68.0° (220).

The phase composition and average crystallite sizes of the studied samples are presented in Table 3. It should be noted that the presented results refer to the crystalline phase. For all the samples, the percentage of the anatase and rutile phase was noticed together with the increase in the CuO percentage. It was found that the ratio of anatase to rutile did not differ significantly, which might be due to the high stability of the crystallite structure of P25 itself. The average crystallite size of anatase does not change and equaled 23 or 24 nm for all the studied materials. The average crystallite size of rutile was also similar for most the investigated samples, and ranged from 38 to 41 nm. The obtained results suggest that solvothermal modification of commercially available titanium dioxide with copper(II) nitrate did not affect the crystallite size of both anatase and rutile phases due to the relatively low temperature during microwave treatment (~250 °C). An increasing tendency for the average crystallite size of copper oxide was observed. For the sample with the lowest copper content, the crystallite size was 23 nm, while for the sample with the highest copper content, it was 45 nm. Only for the P25-R-Cu-8.0 and P25-R-Cu-10 samples were slightly lower values noticed, and these were 37 and 35 [nm], respectively.

#### 2.1.4. Helium Density Measurements

**Figure 3:**
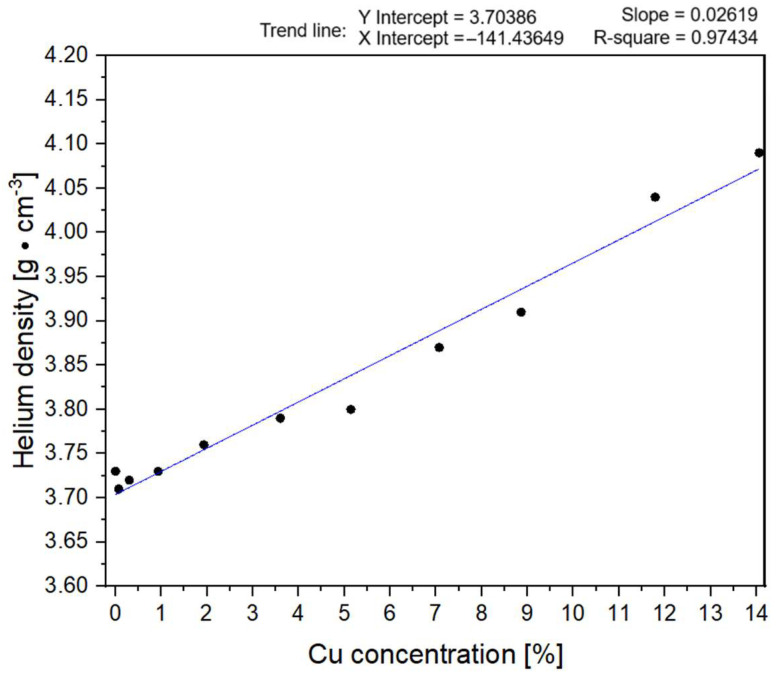
Helium density measurement values in relation to ICP-OES-measured copper concentration.

The measured density values are presented in Figure 3. It was observed that modification with suspension of ammonic copper(II) nitrate in a microwave-assisted solvothermal reactor allows for the density change of the samples. This is most likely due to both the presence of copper(II) oxide and the occurrence of nucleation processes of its particles and amorphous phases of P25 in the presence of an alcohol–water environment in an MW solvothermal reactor [58,59].

On the microscopic scale, microwaves increase the friction between the molecules which is a result of frequent breaking and reforming hydrogen bonds, which transforms MW energy into heat (E_MV_ = Q). This additional heat can change the structure of the solvent hydrogen bonds, which in turn may cause different dynamics of a reaction [60].

The rate of nucleation, J (the number of nuclei formed per unit of volume per unit time [m^−3^ · s^−1^]), can be expressed by the following Arrhenius Equation:

(1) $$J=A\cdot \exp\left(-\frac{\Delta G_{\mathrm{crit}.n}}{k_{B}T}\right)$$

where A is a pre-exponential factor or reaction frequency, ΔG_crit.n_ is the Gibbs free energy barrier for the formation of critical nuclei, k_B_ is the Boltzmann constant and T is the absolute temperature in Kelvins [61].

Mathematically, it can be proven that when the absolute value of temperature increases, the overall value of J also increases due to the $\exp\left(\frac{\Delta G_{\mathrm{crit}.n}}{k_{B}T}\right)$ fragment having simultaneously lower value and being in the denominator of the equation.

Hence, microwave-assisted solvothermal heating provides a higher nucleation rate, which makes it more probable for TiO_2_ nanoparticles to aggregate and/or agglomerate. Those additional nucleation processes lower the volume of the overall material and, in turn, raise the overall density of a material.

The addition of ammonic copper(II) nitrate suspension during the solvothermal processing of the P25 photocatalyst allows for a further increase in sample density, with the highest value of 4.09 g·cm^−3^ reached for the sample P25-R-Cu-14. The general trend observed in values is that an increase in copper concentration is positively correlated with measured density values. This occurrence can be considered an additional confirmation of successful copper addition—since the measured densities of samples are indeed, as expected, higher than the density of reference P25-R-NH_3_ (3.73 g·cm^−3^) due to the larger presence of copper in the sample confirmed by previously described methods.

#### 2.1.5. FESEM Studies and EDS Studies

**Figure 4:**
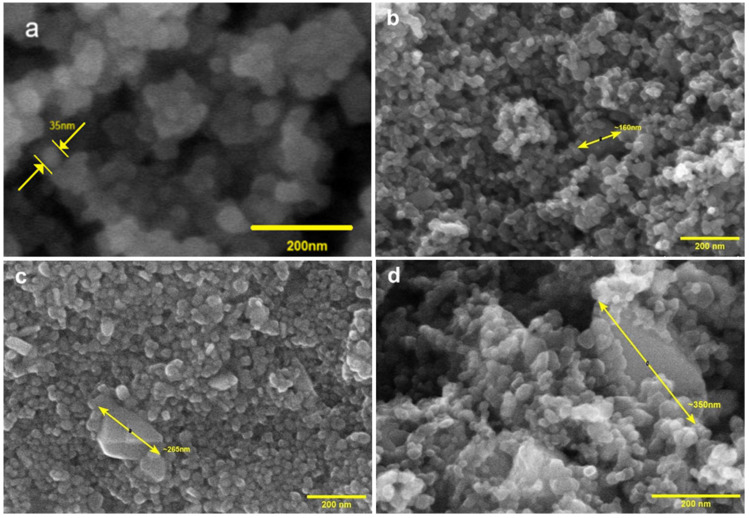
Example SEM images of the investigated materials: (**a**) P25-R-NH_3_, (**b**) P25-R-Cu-0.5, (**c**) P25-R-Cu-4.5, (**d**) P25-R-Cu-12.

Scanning electron microscopy with field emission (FESEM) was performed for the researched samples. SEM imaging of the reference sample, P25-R-NH_3_—Figure 4a—showed the occurrence of irregular and rounded structures of titania with dimensions ranging from 10 to 40 nm, without being able to distinguish whether they were anatase or rutile. Titania materials showed a tendency to create agglomerates with visible boundaries. Additionally, it can be observed that agglomerates of various forms of titania are usually connected—this close coexistence can be attributed to the presence of an amorphous phase between the anatase and rutile phases [62,63].

Modification with copper(II) nitrate in the studied range (theoretical value of max. 14 wt.%) did not affect the size or shape of the observed titania structures (Figure 4b–d). Copper(II) oxide is present in the samples in the form of structures with shapes and sizes significantly different from those of TiO_2_; the observed size was even above 350 nm (Figure 4d).

Knowing that the estimated average crystallite size of copper(II) oxide does not exceed 45 nm and the microwave-assisted solvothermal reactor promotes the nucleation processes, it can be concluded that CuO is present predominately in aggregate form, where the boundaries of individual crystallites are not observed. This observation is further proven by the literature review, where it is described that the larger amount of Cu^2+^ ions present in the system increases the tendency of CuO nanoparticles to nucleate and, further, to aggregate [64,65]. This tendency has been observed in this work and other works [64,66].

The copper content determined by the energy-dispersive spectroscopy (EDS) method shown in Table 1 shows discrepancies, especially in the concentration range above 5 wt.%, with the projected amount of copper physically introduced into the samples confirmed by the ICP-OES method. This is reasonable when one considers that in EDS analysis the surface region is examined, whereas ICP-OES determines the total concentration in the bulk/volume. High RSD values indicate point (uneven) enrichment of titania with copper, which may be related to the occurrence of CuO in the form of large aggregates.

#### 2.1.6. Brunauer, Emmett and Teller (BET) Specific Surface Area Measurements

**Figure 5:**
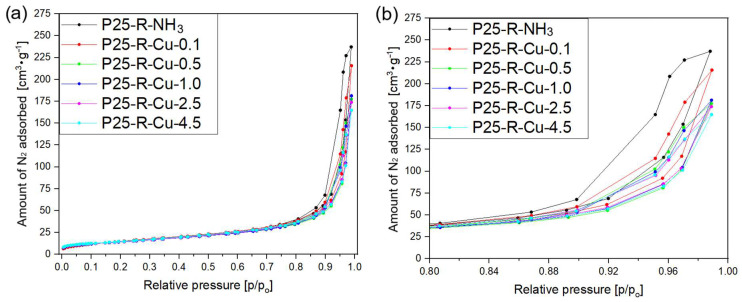
N_2_ adsorption/desorption isotherms of the commercial titania photocatalyst P25 modified with various amounts of copper(II) nitrate (up to 4.5 wt.% theoretical): (**a**) full isotherms, (**b**) magnification of the hysteresis loops.

**Figure 6:**
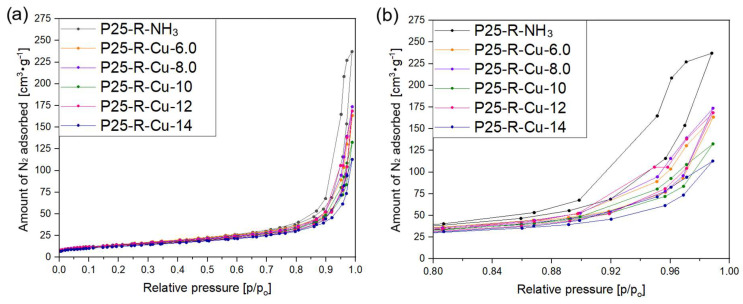
N_2_ adsorption/desorption isotherms of the commercial titania photocatalyst P25 modified with various amounts of copper(II) nitrate (above the 4.5 wt.% theoretical): (**a**) full isotherms, (**b**) magnification of the hysteresis loops.

In order to investigate the textural properties of the obtained samples, low-temperature nitrogen adsorption/desorption measurements were carried out and the resulting isotherms are presented in Figure 5 and Figure 6. The obtained isotherms are very similar to the ones obtained by Yu et al. [67]—their shape can be considered a type II isotherm according to the IUPAC classification [68,69].

Type II isotherms are characteristic of nonporous or macroporous materials, where the shape is attributed to the formation of unrestricted monolayer–multilayer adsorption up to high p/p_o_ values [68]. Figure 5a,b and Figure 6a,b present isotherms of samples modified with copper(II) nitrate in relation to the reference sample (P25-R-NH_3_). The shapes of isotherms show that the final volumes of adsorbed N_2_ (V_total_) at a relative pressure of p/p_o_ = 0.99 were decreasing as the amount of copper introduced to the sample was higher. This is most likely due to the accelerated process of nucleation of the existing clusters on the hot spots at the solid–liquid interfaces were caused by fast microwave heating [65], which formed additional hindrances to the pores resulting in a lower adsorptive capacity of the samples. These effects also often result in the formation of additional structures, which are suggested to be responsible for lower amounts of nitrogen adsorbed.

H4-type hysteresis loops were noticed for all the samples, which is characteristic of the materials consisting of non-rigid aggregates of particles [68] and characteristic of the materials where macropores are not completely filled with pore condensate. As the weight amount of copper in the sample increases, loops for P25-R-Cu-X samples becoming both smaller and placed lower (compared to the reference material). The smallest hysteresis loop was found in the P25-R-Cu-14 sample, which suggests that the lower content of total pores in the modified samples is a result of them becoming blocked with the increased amount of copper(II) oxide present in the sample [70,71].

Based on the nitrogen sorption isotherms, the specific surface areas and total pore volumes were investigated and the obtained values are presented in Table 4. The specific surface area of reference sample P25-R-NH_3_ reached 52 m^2^·g^−1^. Regardless of the concentration of copper(II) cations used for modification, the values of the surface area of all modified samples are similar and range from 45 to 56 m^2^·g^−1^, and the highest value of the S_BET_ area was calculated for P25-R-Cu-0.1% (56 m^2^·g^−1^). Samples with a copper concentration above 6 wt.% (theoretical) show a slight trend of declining S_BET_ values as the amount of copper present in the preparation of a sample is raised—that observation is similar to the other research reported in the literature [72].

The investigation of reference sample P25-R-NH_3_ revealed its high pore volume, at a level of 0.37 cm^3^·g^−1^. Modification of the commercial P25 with copper(II) ions led to the gradual diminishment of the pore volume of materials with the increase in copper concentration in samples. Thus, the sample with the lowest content of copper, P25-R-Cu-0.1, expressed a total pore volume value of 0.33 cm^3^·g^−1^, whereas pore volume for the sample P25-R-Cu-14 (with the highest content of copper) was measured to be 0.18 cm^3^·g^−1^. This decrease in total pore volume as well as the surface area of the prepared catalysts is mainly due to pore blocking by the CuO present in the system [73].

### 2.2. Measurements of Photocatalytic Activity

#### 2.2.1. Photocatalytic Activity Under UV-Vis Lamp

**Figure 7:**
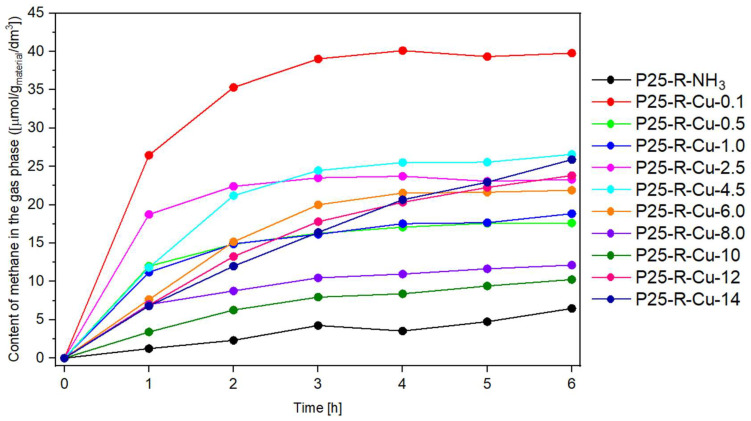
The content of methane obtained in the experiment over time.

The results of a photocatalytic reduction of carbon dioxide to methane are shown in Figure 7. All samples modified with copper(II) nitrate allowed us to obtain significantly more micromoles of methane as compared to the reference material. Most of the samples (characterized by an ICP-OES-measured copper concentration lower than 7.07 wt.% Cu for P25-R-Cu-8.0) are characterized by a quick increase in product in the gas phase during the first two hours of the process and then reaching a plateau. Samples with the highest concentrations of copper (most notably P25-R-Cu-12 and P25-R-Cu-14) present a slower but more consistent increase in values.

The highest value of a product was measured for the sample P25-R-Cu-0.1 (0.07 wt.% Cu). This observation, coupled with the likely incorporation of Cu^2+^ particles to the amorphous structure TiO_2_ [74] described earlier, suggests that the incorporation of larger Cu ions into the TiO_2_ structure may create additional oxygen vacancies. It has been reported that oxygen vacancy can improve the photocatalytic activity of TiO_2_ by reducing the recombination rate between electrons and holes and adding an additional electron level below the conduction band [75].

According to Nian et al. [76], the successful introduction of Cu^2+^ into the TiO_2_ structure tends to be higher, especially for low amounts of Cu doping [66,76].

**Figure 8:**
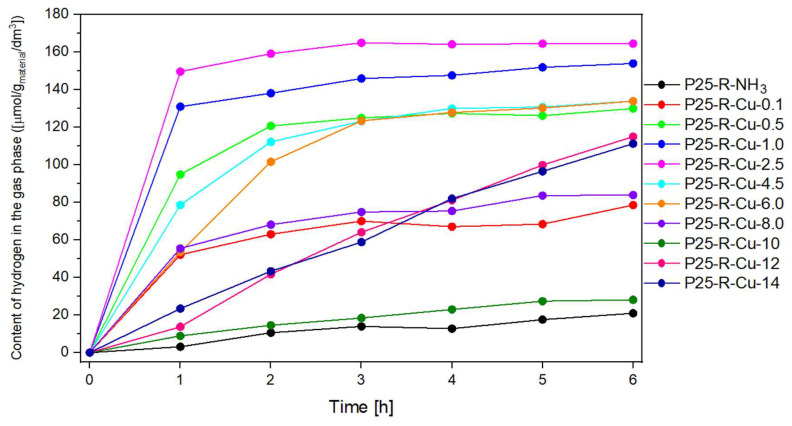
The content of hydrogen obtained in the experiment over time.

Comparing the values representing the content of hydrogen measured in the gas phase (Figure 8) with the curves representing the amount of methane in the gas phase, multiple similarities can be observed. Firstly, it can be seen that modification of P25 with copper(II) nitrate enhances the photocatalytic activities of all the samples in comparison to the reference sample (P25-R-NH_3_). Secondly, the shapes of hydrogen graphs tend to repeat the shapes of methane graphs (with samples up to P25-R-Cu-8.0 being characterized by a plateau).

The highest value of obtained hydrogen was achieved for sample P25-R-Cu-2.5, which could be attributed simultaneously to a lowered band gap energy of a material (Eg = 2.96 eV) (via both the incorporation of Cu^2+^ to the material and its coexistence with CuO [50]) and raising the Fermi level of a semiconductor photocatalyst, which allows one to reach the highest occupied molecular orbital (HOMO) level of the adsorbed molecule [77].

The likely mechanism of the process indicates that photoexcited electrons migrate to the surface of the photocatalyst and are trapped by the CuO nanoparticles present in a sample because the Fermi energy level of CuO is lower than that of TiO_2_ [45,66]. In that scenario, copper(II) oxide acts as a co-catalyst [41].

Another important effect of Cu incorporation is its role as an electron scavenger. It has been reported that the addition of Cu into TiO_2_ enhances its activity by creating a new energy level below the conduction band of TiO_2_, which can act as an electron trapper [34]. This trap can further separate photogenerated electron–hole pairs, resulting in lowering the overall recombination rate; therefore, more electrons and holes can further be involved in redox reactions [34,78]. It is likely that both of the described mechanisms are responsible for enhancing the photoactivity of the samples.

From comparing both the methane and hydrogen graphs, it can be concluded that within the group of plateaued graphs, samples with higher concentrations of Cu tend to present lower photocatalytic activity as compared to the ones with lower loadings of Cu.

This phenomenon can be explained due to an excess amount of Cu present in those samples. It has been reported that an excess amount loading of a metal (acting as an electron scavenger) leads to a decrease in photocatalytic performance due to the covering of active centers (most likely on the P25:Cu interfaces) by the agglomerated metal compound particles (most dominantly CuO) [66]. This blocking results in less light being absorbed and fewer charge carriers being formed. Therefore, the optimum level of added metal is needed to maximize the photocatalytic activity [34]. In our studies, it was found that different values of copper loading are optimal for obtaining different products—the optimal copper concentration for obtaining methane was 0.07 wt.% Cu (P25-R-Cu-0.1), whereas for obtaining hydrogen, it was 1.93 wt.% Cu (P25-R-Cu-2.5).

**Figure 9:**
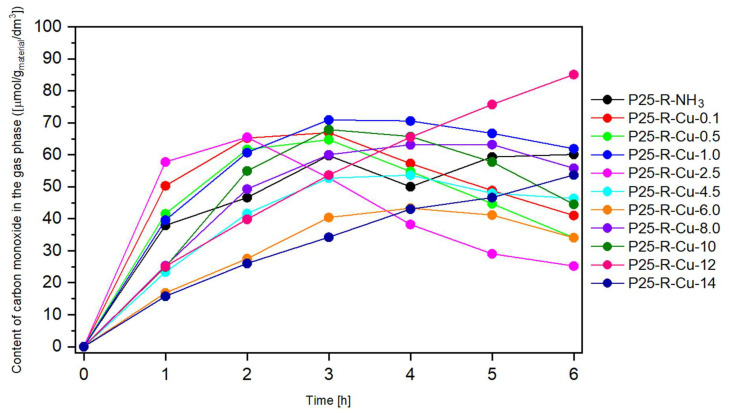
The content of carbon monoxide obtained in the experiment over time.

In the case of graphs presenting amounts of carbon monoxide measured in the system (Figure 9), the results are mostly comparable with the performance of the reference sample. Most of the samples exhibit a decline in carbon monoxide production during the second or third hour of measurement. However, for the samples with higher copper loads (such as P25-R-Cu-6.0 and P25-R-Cu-8.0), the beginning of a decline can be observed in the later stages of measurement, which suggests that more linear graphs of P25-R-Cu-12 and P25-R-Cu-14 would also bend during the longer process. These observations suggest that carbon monoxide may be used in competing reactions [8]. Copper is generally known as a monometallic element that can convert CO_2_/CO into C_2+_ hydrocarbons and that oxygenates with appreciable activity and selectivity [79,80,81], so the shape of the graph may indeed be explained by CO being used in further reduction in the reactor, leading to products which were not measured in this study.

#### 2.2.2. Selectivity Calculations

Using those reactions as possible parallel pathways for carbon dioxide conversion, the selectivity of this process can be calculated for each of the described photocatalysts (Table 5).

### 2.3. Selectivity of Carbon Dioxide Conversion for the Described Samples

Samples modified with copper tend to present significantly higher selectivity of CO_2_ conversion towards methane than the reference sample (P25-R-NH_3_). This occurrence is in line with the literature, which often claims that CO is considered an intermediate product of carbon dioxide being reduced to hydrocarbons [82,83]. The incorporation of copper into the sample allows one to accommodate additional steps of reductive conversion, which shifts the values of selectivity in this process to the benefit of methane.

The highest value of CO_2_ conversion towards methane was calculated for the sample P25-R-Cu-0.1 (82.5%), which may suggest that the introduction of small amounts of copper to the samples can be more beneficial than modifications using higher Cu^2+^ concentrations. No clear trend correlation between the described selectivity towards methane and copper concentration may indicate that the form of copper present in the material lowers the amount of total active sites available for the processes. In the described samples, copper is primarily found in the form of rather agglomerated CuO crystallites, which may block a certain number of active sites. That possibility, in return, lowers the chance of attributing a reliable trend to the changes observed in the data. The addition of a smaller amount of copper may reduce the possibility of agglomeration and, therefore, be beneficial overall for methane formation.

### 2.4. Selectivity Relative to the Total Amount of Hydrogen Present in the Gas Phase

When basing the selectivity calculations on the total amount of hydrogen present in the gas phase, the values of selectivity shift significantly toward the hydrogen formation.

Similarly to the carbon dioxide conversion-based calculations, the reference sample presents a reversed profile of selectivity as compared to the samples modified with copper ions (meaning that selectivity towards carbon monoxide is the highest and selectivity towards hydrogen is the lowest as opposed to the rest of the samples).

Additionally, upon interpreting Table 5, it can be observed that with the higher weight concentration of copper cations used in P25-TiO_2_ modification, the selectivity towards all products tends to be comparable.

## 3. Experimental Section

### 3.1. Material Preparation

The materials researched in this article were prepared by processing an ammonic P25 and copper(II) nitrate suspension in a microwave-assisted solvothermal reactor. The obtained samples were named in the convention of P25-R-Cu-X, where R means that a sample was processed in the microwave-assisted reactor and the value X in the name of the sample refers to the mass concentration of Cu^2+^ cations present in the dry mixture of P25 + Cu(NO_3_)_2_ used to process a sample during a solvothermal process. In this study, Evonik (Degussa) Aeroxide^®^ P25 titania was used. This is due to the significant content of the amorphous phase [74,84] and the presence of a phase junction formed between the surface anatase nanoparticles and rutile particles that may facilitate the transfer of the photogenerated charge carriers and prevent the electron–hole recombination [62,71]. P25-R-NH_3_ is considered a reference sample—it was processed in a microwave-assisted solvothermal reactor with the addition of ammonia water (to pH = 10).

Suspensions used in the preparation of the photocatalysts were prepared by mixing 2 g of P25 (AEROXIDE^®^ TiO_2_ P25, Evonik Industries AG, Essen, Germany), a mass of copper(II) nitrate corresponding to a specific copper concentration, and 200 mL of a mixture consisting of 96% ethanol and water in a volumetric ratio of 1:1. Materials were sonified for 20 min to obtain a well-dispersed suspension. Next, the mixture was transferred to a magnetic stirrer, and ammonia water (25% NH_3_∙H_2_O) was added until the pH of the mixture was stabilized at pH = 10. After 60 min of mixing, processing in a solvothermal microwave-assisted reactor was carried out. Suspensions were placed in a Teflon container and inserted into the reactor (Magnum II, Ertec-Poland Edward Reszke, Wrocław, Poland), in which a pressure of 40 bar was maintained for 15 min. After the treatment in the reactor, the samples were dried at 80 °C until they became grayish or brownish powders (the more copper concentration was used, the darker the hue was). Powders were then ground in an agate mortar to a uniform consistency and subjected to washing. The washing was continued until the filtrate was transparent and its pH was close to neutral. The neutralized powders were dried, then ground again to a uniform consistency, and transferred into a plastic container. The visual representation of the samples is presented in Table 2.

### 3.2. Material Characterization

#### 3.2.1. ICP-OES (Inductively Coupled Plasma) Spectroscopy Measurements

To determine the content of Cu in the tested samples, inductively coupled plasma with optical emission spectrometry was used (ICP-OES, Perkin Elmer Avio 500, Waltham, MA, USA).

#### Preparation of Samples

Before ICP-OES analysis, 0.1 g of each sample was weighed and transferred to each of the Erlenmeyer flasks. Then, 6 g of (NH_4_)_2_SO_4_ and 20 mL of concentrated (98 wt.%) H_2_SO_4_ solution were added to each of the flasks. Acidic suspensions of samples were heated until the precipitate dissolved, which, depending on the copper content in the modified samples, formed a slightly yellow or blue solution. After the flasks had cooled to approximately room temperature, 120 mL of deionized water was carefully added to each flask. The solutions were mixed and then transferred to the 250 mL volumetric flasks and made up to the mark with distilled water. The samples prepared in this way were filtered and subjected to ICP-OES analysis, which is described below.

#### ICP-OES (Inductively Coupled Plasma) Spectroscopy Measurements

Modified samples based on TiO_2_ were analyzed for copper content by inductively coupled plasma optical emission spectrometry (ICP-OES). To prepare the calibration solutions, a Cu standard solution (Merck KGaA Corporation, Darmstadt, Germany) at a concentration of 1000 µg/mL in 5% HNO_3_ was used. Furthermore, to prepare the standard solutions, ultrapure water fitting the FP and PN-EN ISO 3696:1999 standards for the first purity class (Hydrolab, Water Purification Systems, Straszyn, Poland) was used in experiments. All the chemicals were used as received without further purification. During the analysis, argon with a purity of 99.999% (Air Liquide, Kraków, Poland) was used as gas. The instrument conditions and determined parameters are given in Table 6.

To estimate the content of copper in the tested samples, the calibration solutions were prepared. This involved dilution of the Cu standard (1000 µg/mL) to the set of weight concentrations allowing us to draw appropriate calibration curves within the range of the expected samples’ copper contents.

#### 3.2.2. UV-Vis/DR Spectra Measurements

The UV–Vis diffuse reflectance spectra of the tested photocatalysts were recorded using a UV-Vis/DR spectrophotometer (V-650, JASCO International Co., Tokyo, Japan) equipped with a PIV-756 (JASCO International Co., Tokyo, Japan) integrating sphere accessory for diffuse reflectance. The spectra were recorded in the 200–800 nm wavelength range. Barium sulfate (BaSO_4_, pure p.a., Avantor Performance Materials Poland S.A., Gliwice, Poland) was used as reference material. The band gap energy (*E*_g_) values were calculated using the modified Kubelka–Munk method described in detail by Peverga et al. [85].

#### 3.2.3. XRD Measurements

The phase composition was determined using the X-ray diffraction method for Cu Kα radiation (λCu Kα = 0.154056 nm) on an Empyrean X-ray diffractometer (Malvern PANalytical B.V., Almelo, The Netherlands). The XRD diffractograms were collected in the 2θ range of 20° to 80° at a step size of 0.02°. The PDF 5+ 2024 International Centre for Diffraction Data database (01-075-2547 PDF4+ card for anatase, 01-079-6029 for rutile and 00-048-1548 PDF4+ card for tenorite) was used for the specification of the phase composition. The mean crystallite sizes of the materials were calculated using the Rietveld method.

#### 3.2.4. Helium Density Measurements

A helium densitometer, ULTRAPYC 1200e (Quantachrome Instruments, Boynton Beach, FL, USA), was used for the materials’ density measurements at 24 ± 1 °C temperature. To guarantee the accuracy of the measurements, the sample vessel was filled to 3/4 of its volume and, before starting the test, the sample was flushed several times with pure helium to desorb other gases.

#### 3.2.5. SEM and EDS Studies

The morphologies of the produced materials were characterized using scanning electron microscopy (SEM). Prior to analysis, all tested samples were immobilized to an alumina round table utilizing carbon tape dedicated to this SEM equipment. Then, the table containing analyzed samples was placed inside the vacuum chamber and degassed under a high vacuum. The analysis was carried out using a SU-8020 (Hitachi Ltd., Chiyoda, Tokyo, Japan) microscope with an acceleration voltage of 5.0 kV at the appropriate magnification to show the structures present in the sample. The X-ray microanalysis, energy-dispersive X-ray spectroscopy (EDS), was performed with an acceleration voltage of 15 kV.

#### 3.2.6. BET Surface Area Measurements

The low-temperature nitrogen adsorption/desorption studies were performed at 77 K using a QUADRASORB evoTM gas sorption automatic system (Quantachrome Instruments, Anton Paar, Austria), together with a MasterPrepTM multi-zone Vacuum and Flow Degasser (Quantachrome Instruments, Boynton Beach, FL, USA), which allowed us to degas samples under a vacuum of 1 × 10^−5^ mbar.

The obtained adsorption/desorption isotherms allowed us to determine the specific surface area (S_BET_) and pore volumes of the obtained materials. The pre-dried samples were then transferred into measuring cells and degassed using MasterPrepTM at 100 °C for 12 h. The Brunauer–Emmett–Teller (BET) equation was used to determine surface areas (S_BET_) in the relative pressure range of 0.05–0.20. The total pore volumes (V_total_) were calculated from the volumes of nitrogen held at the highest relative pressure (p/p_0_ = 0.99).

### 3.3. Photocatalytic Process

#### 3.3.1. Photocatalytic Setup

The gas phase photocatalytic activity experiments were conducted in a glass bottle-shaped reactor with a volume of 766 cm^3^. During the processes, a medium-pressure mercury lamp (TQ150 Z3, Heraeus, Germany) with a power of 150 W was utilized. It is characterized by a broad range of radiation in both UV and visible light (250–600 nm, maximum 365 nm). The lamp was placed in a quartz cooler and constantly supplied with water at 18 °C through a chiller equipped with a pump (Minichiller 280 OLÉ, Huber, Germany). The reactor and the rest of the system were enclosed in a thermostat chamber to eliminate other light sources and maintain a stable process temperature of 20 °C.

For each process, samples were prepared by spreading the aqueous suspension of photocatalyst onto the glass fiber (FF 45 VLIES 50; 40 g/m^2^), drying it and then placing it inside the reactor along with 10 cm^3^ of distilled water at the bottom of the reactor. The reactor’s interior was then purged with pure CO_2_ (Messer, Chorzów, Poland) for 16 h. After this time, the system was sealed, and the lamp was turned on. The gas was mixed using a pump with a flow rate of 1.6 dm^3^/h during the process. The process was conducted for 6 h, and samples for analysis were collected every 1 h.

The gas phase composition was analyzed using a Master GC chromatograph (DANI Instruments, Italy) equipped with micropacked Shincarbon ST 100/120 column. The analyses were performed using TCD and FID detectors. The methanizer was placed between the detectors in order to convert the inorganic product (carbon monoxide) to methane, allowing us to accurately determine its amount using FID. The carrier gas was argon. The volume of the analyzed gas sample was 1 cm^3^. The amount of hydrogen, carbon monoxide and methane in a gas phase was calculated based on the calibration curve. The setup is analogous to the one presented in the work of Morawski et al. [86].

#### 3.3.2. Selectivity of the Obtained Materials in the Carbon Dioxide Photoreduction Process

The ratio of an amount of reactant used to form a certain product A per the total amount of this reactant consumed during the process can be defined as selectivity towards product A [87], which can be expressed using Formula (2):

(2) $$S_{A}\left[\%\right]=\frac{n_{A}}{n_{\mathrm{So}}{-n}_{\mathrm{Se}}}\cdot 100\%$$

where

S_A_—selectivity of the substance conversion towards the product A;

n_A_—the amount of product A moles measured at the end of the process;

n_So_—the amount of substrate moles before the process;

n_Se_—the amount of substrate moles after the process.

The method used to determine the selectivity of samples is analogical to the description in another article [88]. It assumes that during the photocatalytic process all the reactants are present in the gas phase, so reactions (3) and (4) can be attributed to the formation of carbon monoxide and methane in the gas phase [8]:

CO_2_ + 2H^+^ + 2e^−^ → CO + H_2_O (3)

CO_2_ + 8H^+^ + 8e^−^ → CH_4_ + 2H_2_O (4)

Using those reactions as possible parallel pathways for carbon dioxide conversion, the selectivity of this process can be calculated for each of the described photocatalysts.

Additionally, the described formula can be used to calculate the selectivity values based on the total amount of hydrogen present in the gas phase (the total H_2_ present and/or spent during alternative reaction pathways). Since the H_2_ molecules may also be considered a product of the overall photocatalytic process (due to the parallel water-splitting process), the selectivity of the process towards all products measured in the gas phase (H_2_, CO and CH_4_) can offer an alternative perspective on the photocatalytic process. To approximate how many moles of H_2_ have been used in the formation of CO or CH_4_ (conversion of CO_2_), 2H^+^ + 2e^−^ was considered as a molar equivalent of an H_2_ molecule and allowed to quantify the total amount of H_2_ produced and used during the process.

## 4. Conclusions

In summary, this paper presents the physicochemical characterization and photocatalytic properties of TiO_2_-based materials modified with copper(II) nitrate in different concentrations ranging from 0.07 wt.% Cu (P25-R-Cu-0.1) to 14.06 wt.% Cu (P25-R-Cu-14). The photocatalysts were obtained by microwave-assisted solvothermal modification of commercially available P25 under a temperature of 250 °C and pressure of 40 bar, which allowed us to change their properties.

The influence of the processing conditions and the copper ion concentration used for the photoactivity of CO_2_ reduction towards various products was determined. It was found that solvothermal modification using copper(II) nitrate did not affect the size of anatase and rutile crystallites, but most likely allowed us to modify the properties of the amorphous phase of the material as evidenced by the changed band gap energy values. All of the samples had enhanced photocatalytic activity as compared to the reference sample. The best sample with regard to methane production was P25-R-Cu-0.1 (0.07 wt.%Cu), for which approximately 40 μmol/g_material_/dm^3^ of the product was obtained with a selectivity of CO_2_ conversion of 82.5%. The obtained samples also allowed for significant hydrogen production via water-splitting reactions, which helped the overall photoreduction process—the best sample for this process was P25-R-Cu-2.5, which allowed us to obtain approximately 160 μmol/g_material_/dm^3^, which is about eight times more than the reference material.

## Figures and Tables

**Table 1:** ICP and EDS measurements indicating calculated weight for copper content values.

No.	Sample	Measured Cu Content (ICP) [%]	RSD (ICP) [%]	Measured Cu Content (EDS) [%]
1	P25-R-NH_3_	0.00	----	----
2	P25-R-Cu-0.1	0.07	0.48	0.12 ± 0.07
3	P25-R-Cu-0.5	0.30	0.35	0.42 ± 0.15
4	P25-R-Cu-1.0	0.93	0.26	1.13 ± 0.41
5	P25-R-Cu-2.5	1.93	0.32	2.80 ± 0.78
6	P25-R-Cu-4.5	3.60	0.42	4.36 ± 1.00
7	P25-R-Cu-6.0	5.14	0.66	6.59 ± 1.47
8	P25-R-Cu-8.0	7.07	0.69	14.83 ± 7.27
9	P25-R-Cu-10	8.86	0.40	14.74 ± 1.11
10	P25-R-Cu-12	11.79	0.34	18.88 ± 4.14
11	P25-R-Cu-14	14.06	0.80	19.76 ± 2.52

RSD—the relative standard deviation of the method.

**Table 2:** The band gap energy values of tested photocatalysts.

Sample	E_g_ [eV]	Figure	Sample	E_g_ [eV]	Figure
P25 (unmodified)	3.24	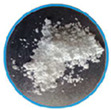	P25-R-Cu-4.5	2.73	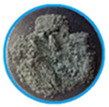
P25-R-NH_3_	3.01	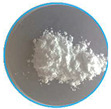	P25-R-Cu-6.0	2.86	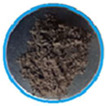
P25-R-Cu-0.1	3.06	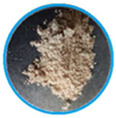	P25-R-Cu-8.0	2.95	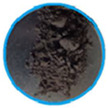
P25-R-Cu-0.5	3.05	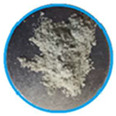	P25-R-Cu-10	2.56	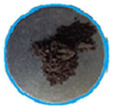
P25-R-Cu-1.0	3.04	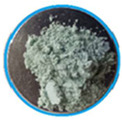	P25-R-Cu-12	2.36	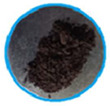
P25-R-Cu-2.5	2.96	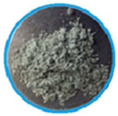	P25-R-Cu-14	2.47	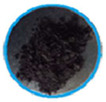

**Table 3:** XRD phase composition and average crystallites size calculated using the Rietveld method.

Sample	Phase Composition [%]			Mean Crystallite Size [nm]		
	Anatase	Rutile	Tenorite	Anatase	Rutile	Tenorite
P25-R-NH_3_	87	13	0	24	38	-
P25-R-Cu-0.1	87	13	0	23	41	-
P25-R-Cu-0.5	88	12	0	23	39	-
P25-R-Cu-1.0	87	13	0	24	40	-
P25-R-Cu-2.5	86	14	1	23	40	-
P25-R-Cu-4.5	84	13	3	23	41	23
P25-R-Cu-6.0	83	12	5	24	38	21
P25-R-Cu-8.0	80	11	9	24	37	35
P25-R-Cu-10	77	11	12	23	35	27
P25-R-Cu-12	73	11	17	24	38	29
P25-R-Cu-14	68	10	22	24	40	45

**Table 4:** Textural parameters of the P25-based modified samples.

Sample	S_BET_ [m^2^·g^−1^]	V_total_ [cm^3^·g^−1^]
P25-R-NH_3_	52	0.37
P25-R-Cu-0.1	56	0.33
P25-R-Cu-0.5	52	0.27
P25-R-Cu-1.0	51	0.28
P25-R-Cu-2.5	51	0.27
P25-R-Cu-4.5	52	0.26
P25-R-Cu-6.0	54	0.25
P25-R-Cu-8.0	51	0.27
P25-R-Cu-10	49	0.21
P25-R-Cu-12	48	0.26
P25-R-Cu-14	45	0.18

S_BET_—specific surface area; V_total_—total volume of nitrogen adsorbed by the sample.

**Table 5:** Calculated selectivity of carbon dioxide conversion towards products measured in the gas phase (after 6 h) for the obtained samples.

	Selectivity (of the CO_2_ Conversion) [%]		Selectivity Relative to the Total Amount of H_2_ in the Gas Phase [%]		
Material	CO	CH_4_	H_2_	CO	CH_4_
P25-R-NH_3_	69.9	30.1	19.6	56.2	24.2
P25-R-Cu-0.1	17.5	82.5	28.9	12.5	58.6
P25-R-Cu-0.5	32.6	67.4	55.4	14.5	30.1
P25-R-Cu-1.0	45.1	54.9	52.8	21.3	25.9
P25-R-Cu-2.5	21.3	78.7	58.1	8.9	33.0
P25-R-Cu-4.5	30.4	69.6	46.7	16.2	37.1
P25-R-Cu-6.0	28.0	72.0	52.4	13.3	34.3
P25-R-Cu-8.0	51.8	48.2	43.8	29.1	27.1
P25-R-Cu-10	52.1	47.9	24.7	39.2	36.1
P25-R-Cu-12	44.0	56.0	37.2	27.6	35.2
P25-R-Cu-14	34.1	65.9	41.4	20.0	38.6

**Table 6:** Instrumental conditions for the ICO-OES measurements.

Parameters	ICP-OES
RF flow rate, W	1400
Plasma gas flow rate, L/min	12
Auxiliary gas flow rate, L/min	0.50
Nebulizer flow rate, L/min	0.80
Pump flow rate, ml/min	1.0
Replicates	5
Delay time, sec	60
Plasma observation	Axial

## Data Availability

Dataset available on request from the authors.
